# Serum concentration of selected macro- and microelements and their correlation with the risk of breast and ovarian cancer among BRCA1 mutation carriers

**DOI:** 10.1186/1897-4287-10-S3-A17

**Published:** 2012-04-20

**Authors:** Magdalena Muszyńska, Grzegorz Sukiennicki, Tomasz Huzarski, Jacek Gronwald, Cezary Cybulski, Tadeusz Dębniak, Aleksandra Tołoczko-Grabarek, Oleg Ashuryk, Anna Jakubowska, Antoni Morawski, Jan Lubiński

**Affiliations:** 1 Read-Gene SA and Pomeranian Medical University Szczecin, Poland

## 

The study was conducted to determine the correlations between serum concentration of selected macro- and microelements, namely: magnesium (Mg), copper (Cu), zinc (Zn), arsenic (As), calcium (Ca), cadmium (Cd) chromium (Cr) and selenium (Se) with increased or decreased predisposition to breast and ovarian cancer.

The subjects selected for the trial were Polish women, positive for at least one of three founder mutations in BRCA1 gene dominating in Poland (5382insC, C61G, 4153delA). Persons with detected tumor were considered as cases and the others were considered as controls. One case and two controls were paired regarding many criteria (e.g. age, family cancer history, cigarettes smoking) to achieve the maximum of similarity between them.

All the elements were quantitatively measured simultaneously in diluted serum samples by inductively coupled plasma mass spectrometry (ICP-MS) using mass spectrometer (Elan DRC-e, PerkinElmer) in standard mode (Mg, Cu, Zn) and in DRC mode (As, Ca, Cd, Cr) with methane as a reaction gas, for removing polyatomic interferences in measurement.

Statistically significant differences have been found for arsenic and zinc. In case of arsenic, individuals classified in the second and fourth quartile had a significantly higher risk of breast or ovarian cancer than those in the first quartile. In case of zinc a significantly lower risk of breast or ovarian cancer was observed among individuals classified in the second quartile. The data are shown in Table 1.

**Table 1:** Arsenic and zinc concentration in each quartiles

As (µg/l)	Cases (n=99)	Controls (n=198)	OR	p-value
1,14 – 3,64[	20(20,2%)	53(26,8%)	1,000	-
[3,64 – 4,51[	29(29,3%)	45(22,7%)	1,708	0.0098
[4,51 – 5,70[	24(24,2%)	51(25,7%)	1,247	0.00719
[5,70 – 57,66	26(26,3%)	49(24,7%)	1,406	0.04843

Zn (µg/l)	Cases (n=99)	Controls (n=198)	OR	p-value

218,89 – 692,48[	31(31,3%)	43(21,7%)	1,000	-
[692,48 – 756,01[	18(18,2%)	56(28,3%)	0,446	0.02709
[756,01 – 838,00[	24(24,2%)	50(25,3%)	0,666	0.07447
[838,00 – 1911,15	26(26,3%)	49(24,7%)	0,736	0.13030

Apart from the influence of single elements some interactions from combinations of arsenic, magnesium, cadmium, zinc and calcium with selenium were also analyzed. The combinations with statistically significant differences between quartiles in the disease risk are shown in Table 2.

**Table 2:** Ratios between analyzed elements and selenium

As/Se	Cases (n=99)	Controls (n=198)	OR	p-value
0.016 - 0.044[	21(21,2%)	53(26,8%)	1,000	-

[0.044 - 0.058[	21(21,2%)	53(26,8%)	1,000	0.163776

[0.058 - 0.072	31(31,3%)	43(21,7%)	1,819	0.000587

[0.072 - 0.14	26(26,3%)	49(24,7%)	1,339	0.005561

				

Mg/Se	Cases (n=99)	Controls (n=198)	OR	p-value

17.5 - 226[	31(31,3%)	43(21,7%)	1,000	-

[226 - 257[	16(16,2%)	58(29,3%)	0,383	0.006663

[257 - 292[	30(30,3%)	44(22,2%)	0,946	0.815701

[292 - 456	22(22,2%)	53(26,8%)	0,576	0.003590

				

Zn/Se	Cases (n=99)	Controls (n=198)	OR	p-value

0.6 - 8.6[	29(29,3%)	45(22,7%)	1,000	-

[8.6 - 9.8[	21(21,2%)	53(26,8%)	0,615	0.217975

[9.8 - 11.1[	22(22,2%)	52(26,3%)	0,656	0.042510

[11.1 - 29.1	27(27,3%)	48(24,2%)	0,873	0.082148

				

Ca/Se	Cases (n=99)	Controls (n=198)	OR	p-value

65.25 - 810[	25(25,3%)	49(24,7%)	1,000	-

[810 - 913[	21(21,2%)	53(26,8%)	0,777	0.976515

[913 - 996[	24(24,2%)	50(25,3%)	0,941	0.112279

[996 - 1638	29(29,3%)	46(23,2%)	1,236	0.017070

